# Quantification of Ploidy in Proteobacteria Revealed the Existence of Monoploid, (Mero-)Oligoploid and Polyploid Species

**DOI:** 10.1371/journal.pone.0016392

**Published:** 2011-01-31

**Authors:** Vito Pecoraro, Karolin Zerulla, Christian Lange, Jörg Soppa

**Affiliations:** Biocentre, Institute for Molecular Biosciences, Goethe-University, Frankfurt, Germany; Health Canada, Canada

## Abstract

Bacteria are generally assumed to be monoploid (haploid). This assumption is mainly based on generalization of the results obtained with the most intensely studied model bacterium, *Escherichia coli* (a gamma-proteobacterium), which is monoploid during very slow growth. However, several species of proteobacteria are oligo- or polyploid, respectively. To get a better overview of the distribution of ploidy levels, genome copy numbers were quantified in four species of three different groups of proteobacteria. A recently developed Real Time PCR approach, which had been used to determine the ploidy levels of halophilic archaea, was optimized for the quantification of genome copy numbers of bacteria. Slow-growing (doubling time 103 minutes) and fast-growing (doubling time 25 minutes) *E. coli* cultures were used as a positive control. The copy numbers of the origin and terminus region of the chromosome were determined and the results were in excellent agreement with published data. The approach was also used to determine the ploidy levels of *Caulobacter crescentus* (an alpha-proteobacterium) and *Wolinella succinogenes* (an epsilon-proteobacterium), both of which are monoploid. In contrast, *Pseudomonas putida* (a gamma-proteobacterium) contains 20 genome copies and is thus polyploid. A survey of the proteobacteria with experimentally-determined genome copy numbers revealed that only three to four of 11 species are monoploid and thus monoploidy is not typical for proteobacteria. The ploidy level is not conserved within the groups of proteobacteria, and there are no obvious correlations between the ploidy levels with other parameters like genome size, optimal growth temperature or mode of life.

## Introduction

The existence of multiple copies of the genome in one cell is called polyploidy. If the genomes originate from several species, the resulting species is allopolyploid, the multiplication of the chromosomes of one species leads to autopolyploidy. Many eukaryotes are polyploid, including species of ciliates, flowering plants, amphibians, fish and even some types of cells in humans. In evolution, the ploidy level can change in both directions, and it has been proposed that the diploid vertebrate genomes were derived by reduction from polyploid ancestors [1]. The advantages and disadvantages of polyploidy have been discussed in several recent reviews [2]–[4]. The advantages are more obvious for allopolyploids, in which alleles of two or more species are combined. They typically outperform their parent strains (heterosis effect). But also autopolyploidy offers advantages, e.g. gene redundancy. Gene redundancy can be accompanied by higher resistance against DNA damaging agents and it offers the possibility of mutating one copy while the wildtype information still remains available.

In contrast to eukaryotes, prokaryotes are usually thought to contain one copy of a circular chromosome. This is typically called “haploidy”, although the term “haploid” does not seem to make much sense in species that do not have a “diploid” stage. The term “monoploid” seems to be more appropriate and thus will be used throughout this paper. It is also used for flowering plants with a C value of one (the C value expresses the haploid complement of the genome from parental contributions) [5].

The best studied bacterial species, *Escherichia coli*, is monoploid when it is grown very slowly, e.g. with a doubling time of 16 hours in a chemostat [6]. When *E. coli* is grown under optimal conditions in the laboratory, the generation time becomes smaller than the replication/segregation time, leading to the re-initiation of replication before the previous replication round had been terminated. The number of replication origins per cell is then larger than the number of termini and the cell becomes merodiploid to mero-oligoploid [7]–[9]. The best-studied gram-positive bacterium, *Bacillus subtilis*, is also monoploid [10], as are several additional species.

However, several bacterial species have been shown to be oligoploid or polyploid, e.g. *Deinococcus radiodurans* and *Borrelia hermsii* [11], [12]. Within the proteobacteria, genome copy numbers have been experimentally determined for *Azotobacter vinelandii* [13]–[15], three *Neisseria* species [16], [17], *Buchnera* spec. [18] and two *Desulfovibrio* species [19]. *N. gonorrhoeae* was revealed to have an average content of three genomes, meaning that it contains two genomes before and four genomes after replication [17]. Therefore, this species is diploid. The genome copy number of *A. vinelandii* highly depends on the growth rate and it has been hypothesized that the polyploid state might be confined to artificially fast growth in the laboratory [13]. *Buchnera* spec. was found to be highly polyploid, but that has been regarded to be exceptional, because this species has a highly reduced genome size and is an endosymbiont [18]. However, also two free-living species of *Desulfovibrio* were found to be oligoploid and polyploid, respectively [19]. A very special case is the symbiosis between *Rhizobium* and galedoid plants, which have evolved the ability to force *Rhizobium* to develop into large, polyploid and unviable bacteriods [20].

Taken together, in stark contrast to the common perception *N. lactamica* and *E. coli* are the only known monoploid proteobacteria, and for *E. coli* this is only true for slow growing cultures. As the number of “exceptions” is meanwhile greater than the species that adhere to the rule, it might be questioned whether monoploidy is really “typical” for proteobacteria. To broaden the insight into the distribution of ploidy levels in different species of proteobacteria we have quantified the ploidy levels for four proteobacterial species, including one species representing epsilon-proteobacteria, which were devoid of members with known ploidy level. A very fast, sensitive and precise Real Time PCR method for the quantification of ploidy levels was used, which had recently been developed to quantify the genome copy numbers of two species of halophilic archaea [21]. We first re-determined the number of origins and termini per cell for fast-growing as well as for slow-growing *E. coli* cultures. After that, the genome copy numbers of three hitherto uncharacterized species were determined. In addition, an overview of proteobacterial species with experimentally-determined ploidy level and selected additional parameters is given, and possible evolutionary advantages of polyploidy are discussed.

## Results

### The Real Time PCR method for ploidy level quantification

**Figure 1:**
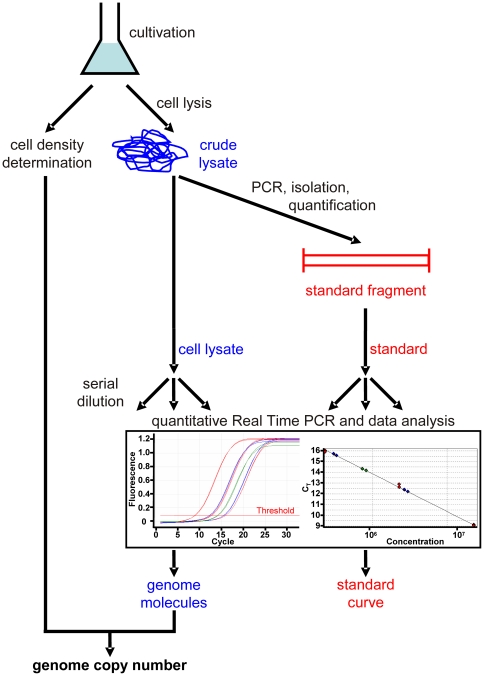
Overview of the method for quantifying ploidy levels. Results from a Real Time PCR experiment are shown including, as templates, serial dilutions of the standard fragment (red curves), the cytoplasmic extract (blue curves) and the cytoplasmic extract with added internal standard (green curves). The horizontal line used for the quantification of the C_t_ values is also included as well as the resulting standard curve.

A scheme of a recently established Real Time PCR approach [21] for the quantification of genome copy numbers is given in Fig. 1. Genomic DNA is used as a template in a conventional PCR reaction to amplify a fragment of about 1 kbp. A dilution series of this fragment is prepared and used for Real Time PCR analysis. A fragment of about 300 bp, internal to the standard fragment, is amplified. The results are used to generate a standard curve. To determine the genome copy number, cells are lysed and a dilution series of the resulting cell extract is analyzed by Real Time PCR in parallel to the standards. The results allow calculating the number of genome copies in the cell extract and, in combination with the cell density of the culture, the ploidy level.

The following points have to be optimized for every new species under investigation and have been optimized for the four species of proteobacteria used in this study: 1) the cell density has to be quantified with a very low variance, 2) it has to be verified that culture growth is exponential and highly reproducible, 3) the method of cell disruption has to be about 100% effective yet leaving the genomic DNA intact, and 4) the Real Time PCR has to be truly exponential, i.e. the differences in C_t_ values of tenfold dilutions of the templates (standard fragment and cell extract) have to be around 3.32. As an additional control that the Real Time PCR efficiencies of standard reactions and “cell extract reactions” were identical the cell extracts were doped with the standard fragment as an internal control and it was verified that the analyses led to the quantification of the respective numbers of added molecules.

One typical example each of a Real Time PCR analysis and a standard curve are included in Fig. 1. For each species, at least three independent cultures were analyzed (and each culture was analyzed at least in triplicates), and average values and standard deviations (sd) were calculated.

### Ploidy of fast- and slow-growing *E. coli* cultures

**Figure 2:**
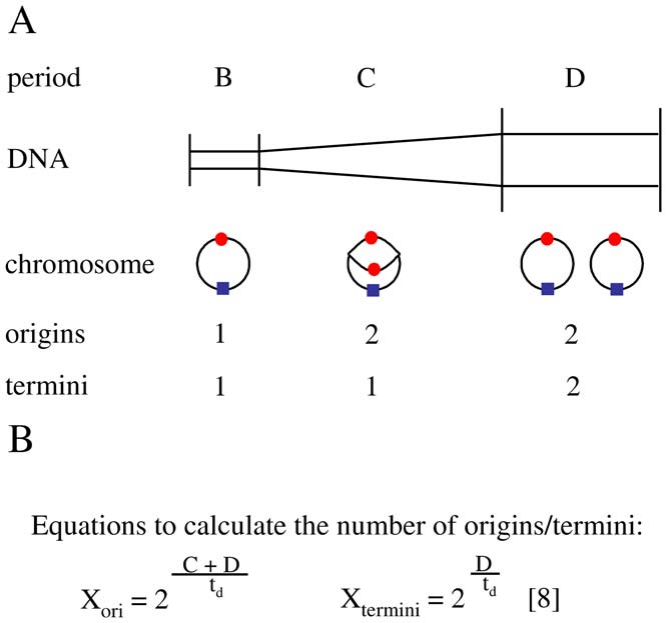
Origins and termini in the cell cycle and in unsynchronized populations. A. Overview of the different phases of the bacterial cell cycle and the number of origins and termini in the different cell cycle phases of a monoploid species. B. Equations to calculate the average numbers of origins and termini in the population.

The first species investigated was *E. coli*, which should serve as a proof-of-principle example that the Real Time PCR method developed for haloarchaea [21], which can be lysed very gently and efficiently by osmotic shock, can also be applied to gram-negative bacteria with a much more rigid cell envelope. Wild type *E. coli* B (DSMZ No. 2840) was chosen to avoid artifacts due to the usage of laboratory strains and to be able to compare the resulting data with results published previously [7], [8]. The cultures were grown in complex medium as well as in synthetic medium with succinate as the sole energy and carbon source, resulting in fast growing cultures with a doubling time of 25 minutes and slow growing cultures with a doubling time of 103 minutes. The respective growth curves as well as the growth curves of all other analyzed species are shown in the Supplementary Material (E. coli: Figures S1 and S2). Care was taken that the pre-cultures used for inoculation were in the exponential growth phase to minimize transition effects. In both cases the copy numbers of two genomic regions were quantified, which represent the intracellular concentration of replication origins and termini, respectively. The results using three independent cultures each for complex and synthetic medium are summarized in Table 1 together with previously published data [7], [8]. As expected, the number of origins was found to be considerably higher in fast growing cultures (6.8±1.6) than in slow growing cultures (2.5±0.4). Also as expected, the difference in the number of termini is smaller with average numbers of 1.7±0.4 termini in fast growing and 1.2±0.3 termini in slow growing cultures. Therefore, fast-growing cells contain on average four times more origins than termini due to the intertwined rounds of replication, while slow growing cells have only twice as many origins as termini. Taken together, the results were in excellent agreement with previous publications (compare Table 1). It should be noted that previous studies did not quantify the origin and termini concentrations experimentally, instead they were calculated using the average DNA content per cell and the length of the C and D period of the cell cycle, which were determined indirectly (an overview of cell cycle periods and the equations used to calculate the origin and termini copy numbers is shown in Fig. 2). Therefore, to our knowledge our results are the first direct quantifications of origin and terminus regions of the *E. coli* chromosome in fast and slow growing cultures.

**Table 1:** Origin and termini copy numbers in *E. coli*.

Culture	Doubling	Cell density	Lysis	No. origins	Average	No. termini	Average
No.	time		effic.	per cell	value	per cell	value
	[hours]	[cells/ml]	[%]	± sd	± sd	± sd	± sd
1	25	8.0×10^8^	96.5	6.9±2.5		1.2±0.1	
2	25	7.7×10^8^	96.4	8.2±1.5	6.8±1.6	2.0±0.2	1.7±0.4
3	25	7.8×10^8^	96.4	5.1±1.1		1.9±0.1	
Ref. [7]	24				6.5		1.9
Ref. [8]	20–20				5.9–9.4		1.5–2.8
4	103	6.5×10^8^	97.3	2.8±0.4		1.0±0.1	
5	103	6.2×10^8^	96.8	2.6±0.5	2.5±0.4	1.1±0.1	1.2±0.3
6	103	6.4×10^8^	96.6	2.1±0.6		1.5±0.2	
Ref. [7]	100				2.0		1.2
Ref. [8]	100				1.6–2.0		1.0–1.3

### Ploidy of Caulobacter crescentus and Wolinella succinogenes

The next species analyzed was *Caulobacter crescentus*, an alpha-proteobacterium. It grew in complex medium with a doubling time of 93 minutes (growth curve: Figure S3). As in cells growing with doubling times around 100 minutes the numbers of origins and termini are more similar than in very fast growing species, only one site of the genome was analyzed, which is located in the middle between origin and terminus. The results are summarized in Table 2. The expected average value for the analyzed region of the genome would be 1.5 for a monoploid species, if the cell cycle parameters of *E. coli* are assumed (a C period of 50 minutes and a D period of 20 minutes). The experimentally-determined value of 2.1 is a little bit higher, but has a high variance (sd = 0.7) and is thus compatible with *C. crescentus* being a strictly monoploid species.

**Table 2:** Genome copy numbers in *C. crescentus*.

Culture	Doubling	Cell density	Lysis	Genomes	Average
	time		effic.	per cell	value
No.	[min.]	[cells/ml]	[%]	± sd	± sd
1	93	5.3×10^8^	94.2	2.3±1.0	
2	93	5.3×10^8^	92.8	1.7±0.7	2.1±0.7
3	93	5.4×10^8^	93.2	2.3±0.4	

The next species analyzed was *Wolinella succinogenes*, an epsilon-proteobacterium, a group without any member with known ploidy level. *W. succinogenes* was grown by anaerobic respiration on formate/fumarate (growth curve: Figure S4). It had a doubling time of 96 minutes, therefore a single genomic region between origin and terminus was analyzed, like for *C. crescentus.* The results are summarized in Table 3. In this case the experimentally determined average value of 0.9 +/− 0.2 is very close to one. The calculated expected value for that genomic location in an exponentially-growing monoploid species is 1.5 if average values of *E. coli* growing at that doubling time are assumed (C period 50 minutes, D period 20 minutes) and 1.2 if a slightly shorter C period and a short D are assumed (C period 40 minutes, D period 6 minutes). Taken together, *W. succinogenes* is a bona fide monoploid species and the experimental results might either be a slight underestimation or might be a first estimation that *W. succinogenes* has a rather short D period.

**Table 3:** Genome copy numbers in *W. succinogenes*.

Culture	Doubling	Cell density	Lysis	Genomes	Average
	time		effic.	per cell	value
No.	[min.]	[cells/ml]	[%]	± sd	± sd
1	96	5.1×10^8^	98.7	0.8±0.2	
2	96	5.0×10^8^	98.8	0.7±0.2	0.9±0.2
3	96	4.9×10^8^	98.6	0.9±0.1	
4	96	5.1×10^8^	98.7	1.1±0.2	

### Ploidy of Pseudomonas putida

The last species analyzed was *Pseudomonas putida*. It was chosen as another free-living species of gamma-proteobacteria and to complement *E. coli* and the polyploid endosymbiont *Buchnera*. *P. putida* grew in complex medium with a doubling time of 46 minutes (growth curve: Figure S5). The levels of the origin region and the terminus region were analyzed separately, like for *E. coli*. The results are summarized in Table 4. *P. putida* turned out to be polyploid, with average values of about 20 origins and 14 termini per cell in the mid-exponential growth phase.

**Table 4:** Origin and termini copy numbers in *P. putida*.

Culture	Doubling	Cell	Lysis	No.	Average	No.	Average
No.	time	density	effic.	origins	value	termini	value
				per cell		per cell	
	[min.]	[cells/ml]	[%]	± sd	± sd	± sd	± sd
1	46	4.8×10^8^	92.5	16.9±0.5		11.6±0.5	
2	46	4.6×10^8^	92.5	22.1±0.8	19.7±2.7	16.3±1.7	13.9±2.4
3	46	4.9×10^8^	92.3	20.1±1.8		13.9±0.7	
Calculated values for three different ploidy levels (with C + D period = 40 + 20 min.):							
oktaploid					19.7		10.8
nonaploid					22.1		12.2
dekaploid					24.6		13.5

## Discussion

### The Real Time PCR method for the quantification of genome copy numbers

The Real Time PCR method for the quantification of genome copy numbers has recently been established to determine the ploidy levels of two species of halophilic archaea, both of which were found to be highly polyploid [21]. Next, it was applied to quantify the genome copy numbers of two species of methanogenic archaea. One species was oligoploid or polyploid, depending on the growth rate, while the other had with about 50 genome copies the highest ploidy level found for any archaeon [22]. A newly established method must be validated against established methods, and that was done in both cases. The ploidy levels of haloarchaea were also determined by quantitative Southern blotting, and the genome copies of methanogenic archaea were also quantified using a spectroscopic method, and in all cases the results of the independent method were in excellent agreement with the results of the Real Time PCR method [21], [22]. In the present study the method is for the first time applied to determine the ploidy levels of bacteria. To validate the method a third time, *E. coli* was chosen as the first species, because this is the best studied bacterial species and the genome copy numbers of slow and fast growing cultures have often been determined using FACS analysis or radioactive labeling of the genome. As discussed below, the results of the Real Time PCR method are again in excellent agreement with previous results obtained with other methods.

### Ploidy of slow and fast growing *E. coli* cultures

The following results obtained with slow and fast growing *E. coli* cultures (Table 1) concur with the expectations: 1) the number of origins is higher than the number of termini, 2) this difference is higher in fast than in slow growing cultures, 3) the number of origins is much higher in fast than in slow growing cultures. The quantified values are in agreement with previously published results (Table 1) [7], [8]. A survey through the relevant literature indicates that the DNA content of *E. coli* is considerably variable. One cause of variance are differences between various *E. coli* strains, and the B/r strain used in many previous studies was even subdivided into three sub-strains “B/r A”, “B/r F” and “B/r K”, because it was observed that “stocks maintained in different collections possessed distinctly different cell division properties” [8]. Another reason for variability is that batch cultures are not in steady state concerning the DNA content even if the dilution of the inoculum is 1000fold and the optical density increases exponentially. It has been reported that the DNA content decreases from early to middle exponential phase, and this had been verified for several strains and for complex as well as synthetic medium [9], [23]. Even the history of the culture has an effect on the ploidy level. After growth in complex medium, nearly all stationary phase cells have two chromosomes, while about 60% of stationary phase cells harbor two chromosomes after growth in synthetic medium with glucose. In contrast, 80% of the cells contained a single chromosome after growth in synthetic medium with acetate [23]. However, regardless of the variability of the absolute values of origins and termini all previous studies showed that the numbers of origins and termini is highly influenced by the growth rate. Fast-growing cells of *E. coli* contain several origins and roughly two termini and are thus not monoploid but merodiploid or mero-oligoploid. Cells with a doubling time of about 100 minutes contain on average about two origins and are also no bona fide monoploids, and only very slowly growing cells of *E. coli* conform to the textbook view and are true monoploids.

### Ploidy of *C. crescentus*, *W. succinogenes* and *P. putida*

*C. crescentus* was found to be monoploid. This meets the expectation based on the life-style of *C. crescentus*, a species with an asymmetric division producing a stalked cell and a swarmer cell, which have different developmental programs. While no direct quantifications of the ploidy level of *C. crescentus* is available, a large number of studies concentrating on its unusual life style have been performed and strongly indicated that *C. crescentus* is monoploid [24]–[26]. Therefore, our results are in congruence with published data.

The epsilon-proteobacteria were devoid of a species with experimentally-determined genome copy number. *W. succinogenes* was chosen because the genome sequence is known, genetic methods have been established, and the species is under active investigation (177 hits in PubMed with “Wolinella succinogenes” in Title/Abstract). *W. succinogenes* is mostly used to study anaerobic energy metabolism [27]. It was shown to be monoploid. The investigation of more species is required to determine whether monoploidy is typical for epsilon-proteobacteria or whether this group also contains oligoploid and polyploid species, like other groups of proteobacteria (see below).

The gamma-proteobacterium *P. putida* was chosen to complement the results obtained with *E. coli* and *Buchnera* and analyze a second free-living species. It was revealed that *P. putida* is polyploid and contains on average about 20 origins and 14 termini. The length of the cell cycle phases are not known, therefore it is not straightforward to compare the experimental results with theoretical expectations. If the values determined for *E. coli* [8] are applied, than for a culture growing with a doubling time of 46 minutes a C period of about 40 minutes and a D period of about 20 minutes can be assumed. The experimental results would then best fit if *P. putida* would be nonaploid, which would lead to calculated average origin and termini concentrations of 22.1 and 12.2 in an exponentially-growing population (Table 4). In summary, *P. putida* was shown to be polyploid, and thus polyploidy in the group of gamma-proteobacteria is not confined to endosymbionts like *Buchnera* with a reduced genome, as has been assumed earlier [18], but also occurs in free-living species.

### Overview of ploidy levels in different species of proteobacteria

An overview of all 11 proteobacterial species with experimentally determined ploidy level is given in Table 5. Several additional parameters were tabulated to allow the detection of possible correlations, i.e. with growth temperature, doubling time, genome size and life style (pathogen or symbiont). However, no correlation of genome copy number to any of these parameters could be detected, e.g. polyploid species either have a very small or a rather large genome (0.66 versus 6.18 Mbp), are symbionts or free-living, and have doubling times of 46 or 2400 minutes.

**Table 5:** Overview of proteobacterial species with experimentally determined ploidy level and selected parameters.

Group	Species	Growth	Doubling	Genome	Pathogen/	Average	Ploidy	Reference
		temperature	time	size	symbiont	genome		
		[^o^C]	[min.]	[Mbp]		copy No.		
α	*Caulobacter crescentus*	30	93	4.01	−/−	2.1	monoploid	this study
	*Azotobacter vinelandii*	30	slow growth	4.7	−/−	1 (−8)	mono-/oligopl.	[13]
	*Azotobacter vinelandii*	30	Fast growth	4.7	−/−	40	polyploid	[13]
β	*Neisseria gonorrhoeae*	37	60	2.15	+/−	3	diploid	[17]
	*Neisseria lactamica*	37	80	∼2.2	−/−	2	monoploid	[16]
	*Neisseria meningitides*	37	40	2.15	+/−	4	diploid	[16]
γ	*Buchnera* spec.	-	-	0.66	−/+	120^a^	polyploid	[18]
	*Escherichia coli*	37	103	4.62	−/−	2.5/1.2^b^	monoploid	this study
	*E. coli*	37	100	4.62	−/−	2.0/1.2^b^	monoploid	[7]
	*E. coli*	37	25	4.62	−/−	6.8/1.7^b^	merooligoploid	this study
	*E. coli*	37	24	4.62	−/−	6.5/2.0^b^	merooligoploid	[7]
	*Pseudomonas putida*	30	46	6.18	−/−	20/14^b^	polyploid	this study
δ	*Desulfovibrio gigas*	28	-, 2400	1.63	−/−	9, 17^c^	polyploid	[19]
	*Desulfovibrio vulgaris*	28	2400	1.72	−/−	4	oligoploid	[19]
ε	*Wolinella succinogenes*	37	96	2.11	−/−	0.9	monoploid	this study

a the genome copy number is highly variable, depending on developmental stage and morph of host [33].

b No. origins/No. termini.

c 9 genomes for ammonia-limited chemostat cultures, 17 genomes for fast-growing batch cultures [19].

Only three to four of the eleven species are truly monoploid, thus it seems that monoploidy is not typical for proteobacteria. Ironically, the textbook example of a monoploid bacterium, *E. coli*, is merooligoploid during fast growth and becomes a bona fide monoploid only upon extremely slow growth [6]. It has been found for additional species that the ploidy level is (directly or indirectly) correlated with growth rate, e.g. *Desulfovibrio gigas* has 17 genome copies during fast growth in batch cultures and 9 genomes per cell in chemostat cultures [19], *Azotobacter vinelandii* becomes highly polyploid at late exponential phase in complex medium (>40 genome copies), but not in synthetic medium (one or a few genome copies) [13], and *Methanosarcina acetivorans* contains 17 genome copies when grown with a doubling time of six hours and 3 genome copies when grown with a doubling time of 49 hours [22]. For several species it has been described that also the growth phase has an influence on the ploidy level. The genome copy number is typically lower in stationary phase than in exponential phase, but counterexamples also exist. Further experiments are needed to reveal whether regulation of the ploidy level in response to environmental conditions and/or to growth phase are widely distributed and typical for prokaryotes.

In summary, the ploidy level is not conserved in proteobacteria, and monoploid, oligoploid and polyploid species exist. Different ploidy levels are found within groups of proteobacteria and even within one genus (*Desulfovibrio, Neisseria*).

### Possible evolutionary advantages of oligo- and polyploidy

Monoploidy is not the rule, but exceptional not only in proteobacteria, but also other groups of bacteria and in one branch of the archaea, the euryarchaeota (a review by J. Soppa is in preparation). It seems that oligo- and polyploidy have evolved independently in different groups of prokaryotes and, therefore, must have one or several evolutionary advantages that outperform the additional energetic costs. While an in depths discussion cannot be part of this publication and will be presented elsewhere, several possible evolutionary advantages that could apply to different species of proteobacteria shall shortly at least be mentioned:

1. polyploid species can have a higher resistance against conditions that induce double strand breaks (DSBs). In the laboratory, often X-ray irradiation is used to induce DSBs, in terrestric environments the evolutionary advantage is most likely the resistance against desiccation, which also induces DSBs. For the bacterium *D. radiodurans*, which contains 5–8 genome copies, it was shown that the same functions are involved in both the high resistance against radiation and against desiccation [28]. It is long known that *D. radiodurans* can restore intact chromosomes from heavily fragmented chromosomes, and recently it was shown that this is a two-stage mechanism involving a high induction of DNA repair synthesis followed by recombination [29], [30];
2. the rate of spontaneous mutations can be reduced in comparison to monoploid species, as has been described for *H. volcanii* [31];
3. polyploidy offers a mechanism of global regulation of gene expression via regulation of the genome copy number, e.g. in response to environmental changes that influence the growth rate. While this has not yet been addressed experimentally, the differences in genome copy numbers in fast versus slow growing cells of *E. coli* and *Desulfovibrio gigas* indicate that proteobacteria make use of that possibility. In fast growing, merooligoploid *E. coli* the dosage of genes near the origin is considerably higher than of genes near the terminus. Therefore, also the genomic localization of genes has the potential to influence the expression level via gene dosage regulation;
4. another possible advantage is gene redundancy, including the possibility to mutate the genome under unfavorable conditions while keeping the wild-type information in other copies. In model experiments it could be shown that under suitable conditions heterozygous cells can be selected that simultaneously harbor two genomes that differ at one locus. Among the proteobacteria this has shown for *A. vinelandii* [15]. Heterozygous cells under specific selective conditions have also been described for cyanobacteria [32]–[34] and for methanogenic archaea [22] as well as for halophilic archaea (Lange, Zerulla, and Soppa, Mol. Microbiol., in revision). Therefore, it might be that this is widespread in polyploid prokaryotes, although no naturally heterozygous cells have been described until now.

Taken together, several possible evolutionary advantages of oligo- and polyploidy exist for prokaryotes (additional advantages apply for pathogens and very large prokaryotes). Therefore, it seems that polyploidy has evolved independently many times in different groups of prokaryotes, and the driving forces might have been different advantages or different combinations of advantages for different species.

## Materials and Methods

### Bacterial species, media and growth conditions

*Escherichia coli* B was obtained from the german culture collection (DSMZ, www.dsmz.de; strain No. 2840). It was grown either in complex medium (SOB^+^) [35] or in M9 synthetic medium [36] with 0.4% (w/v) succinate as carbon and energy source. 20 ml cultures were grown in 100 ml Erlenmeyer flasks at 37°C with a rotating frequency of 200 rpm.

*Pseudomonas putida* KT2440 was obtained from the german culture collection (DSMZ, www.dsmz.de; strain No. 6125). It was grown in a medium recommended by the german culture collection (www.dsmz.de; medium No. 1). 20 ml cultures were grown in 100 ml Erlenmeyer flasks at 30°C with a rotating frequency of 200 rpm.

*Caulobacter crescentus* was obtained from Urs Jenal (Biocentre, Basel, Switzerland). It was grown in PYE medium [37]. 20 ml cultures were grown in 100 ml Erlenmeyer flasks at 30°C with a rotating frequency of 200 rpm.

*Wolinella succinogenes* (strain with the DSMZ No. 1740) was obtained from Jörg Simon (University of Darmstadt, Germany). It was grown anaerobically in formate/fumarate medium [38]. 50 ml cultures were incubated in 100 ml flasks at 37°C without shaking.

### Growth curves and quantification of cell densities

The cells were pre-grown in exponential phase for at least 50 generations by serial dilutions of pre-cultures before inoculation of the main cultures with an initial density of 10^7^ cells/ml. Cell densities were determined using a Neubauer counting chamber. At least three independent cultures were used for the determination of an average growth curve and the quantification of genome copy numbers by Real Time PCR (see below). The doubling time was determined by fitting a straight line to the half-logarithmic representation of the cell densities in exponential phase. All growth curves are shown in the Supplementary Material (the complete growth curves are shown in direct representation and, in addition, the exponential phase in half-logarithmic representation).

### Preparation of cell extracts

Exponentially growing cultures (50 ml for *W. succinogenes,* 20 ml for the other three species) with a cell density of about 5×10^8^ cells/ml were harvested (20 min, 5000 g, 4°C) and the cells were resuspended in 4 ml or 2 ml of 10 mM Tris/HCl, pH 7.2. Lysozyme was added to a final concentration of 2 mg/ml and the cells were incubated until more than 90% of the cells had been lysed, which was verified by determination of the densities with a Neubauer counting chamber. The integrity of genomic DNA was verified by analytical agarose gel electrophoresis. Aliquots of the cytoplasmic extracts were dialized on membrane filters against aqua bidest. Serial dilutions were generated and 5 µl aliquots were included as template in Real Time PCR analyses for quantification of genome copy numbers (see below).

### Quantification of ploidy levels

To determine genome copy numbers, a recently developed Real Time PCR approach was applied [21]. At first fragments of about 1 kbp were amplified using standard PCRs with isolated genomic DNA as template. The amplified genomic regions are summarized in Table 6. A list of the primers used for these PCRs and of all other primers is available upon request. The fragments were purified by preparative agarose gel electrophoresis and the AxyPrepDNA Gel Extraction Kit (Axygen Biosciences, USA). The DNA mass concentrations were determined photometrically and the concentrations of DNA molecules were calculated using the molecular weights computed with “oligo calc” (www.basic.northwestern.edu/biotools). For each standard fragment a dilution series was generated and used for Real Time PCR analysis in parallel with the dilution series of the respective cell extract. The “analysis fragments” were 200–300 bp and exact sizes and genomic localizations are summarized in Table 6. The Real Time PCR analyses were performed as previously described [21]. A negative control (no template control) was also included. By comparison of the C_t_ differences of the different dilutions it was verified that the PCR was exponential at least up to the threshold DNA concentration used for the analysis (i.e. a tenfold dilution corresponds to a C_t_ difference of about 3.32). A standard curve was generated and used to calculate the genome copy numbers present in the dilutions of the cell extract. As an additional control, defined numbers of the standard fragment were added as internal standards to the cell extract and it was verified that the analysis led to the quantification of the added number of molecules. Two controls were performed that only the desired “analysis fragment” had been amplified during the Real Time PCR: 1) the melting points of all products were determined after each run, and 2) the size of the product and the absence of other products were verified by analytical agarose gel electrophoresis.

**Table 6:** Standard and analysis fragments used for copy number quantifications.

Species	Fragment	Size	Genomic localization
		[nt]	
*Caulobacter crescentus*	standard	1097	1 500 875–1 501 971
	analysis	206	1 501 623–1 501 828
*Escherichia coli*	standard ori	1052	3 924 921–3 925 972
	analysis ori	227	3 925 392–3 925 618
	standard term	875	1 550 252–1 551 126
	analysis term	258	1 550 623–1 550 880
*Pseudomonas putida*	standard ori	1008	3 750 292–3 751 299
	analysis ori	291	3 750 358–3 750 648
	standard term	927	608 936–609 862
	analysis term	209	609 124–609 332
*Wolinella succinogenes*	standard	1036	800 014–801 049
	analysis	219	800 381–800 599

## Supporting Information

Figure S1 **Growth of** ***Escherichia coli*** **in complex medium.** A. Growth of *E. coli* in SOB+ complex medium. Samples for the determination of the genome copy number were taken in the exponential growth phase at a cell density of about 8×108 cells/ml. B. Half logarithmic graph of the exponential growth phase. The line of best fit results in a doubling time of 25 min
(PDF)

Figure S2 **Growth of** ***Escherichia coli*** **in synthetic succinate medium.** A. Growth of *E. coli* in M9 minimal medium with succinate as energy and carbon source. Samples for the determination of the genome copy number were taken in the exponential growth phase at a cell density of about 6×108 cells/ml. B. Half logarithmic graph of the exponential growth phase. The line of best fit results in a doubling time of 103 min.
(PDF)

Figure S3 **Growth of** ***Caulobacter crescentus*** **.** A. Growth of *C. crescentus* in PYE complex medium. Samples for the determination of the genome copy number were taken in the exponential growth phase at a cell density of about 5×108 cells/ml. B. Half logarithmic graph of the exponential growth phase. The line of best fit results in a doubling time of 93 min.
(PDF)

Figure S4 **Growth of** ***Wolinella succinogenes*** **.** A. Growth of *W. succinogenes* in fumarate medium. Samples for the determination of the genome copy number were taken in the exponential growth phase at a cell density of about 5×108 cells/ml. B. Half logarithmic graph of the exponential growth phase. The line of best fit results in a doubling time of 96 min.
(PDF)

Figure S5 **Growth of** ***Pseudomonas putida*** **.** A. Growth of *P. putida* in Nutrient Broth complex medium. Samples for the determination of the genome copy number were taken in the exponential growth phase at a cell density of about 5×108 cells/ml. B. Half logarithmic graph of the exponential growth phase. The line of best fit results in a doubling time of 46 min.
(PDF)
