# Supplementary figures and images for: Quantification of Ploidy in Proteobacteria Revealed the Existence of Monoploid, (Mero-)Oligoploid and Polyploid Species
Source: PLoS One. 2011 Jan 31;6(1):e16392. doi: 10.1371/journal.pone.0016392 (PMC3031548; doi:10.1371/journal.pone.0016392)

**A**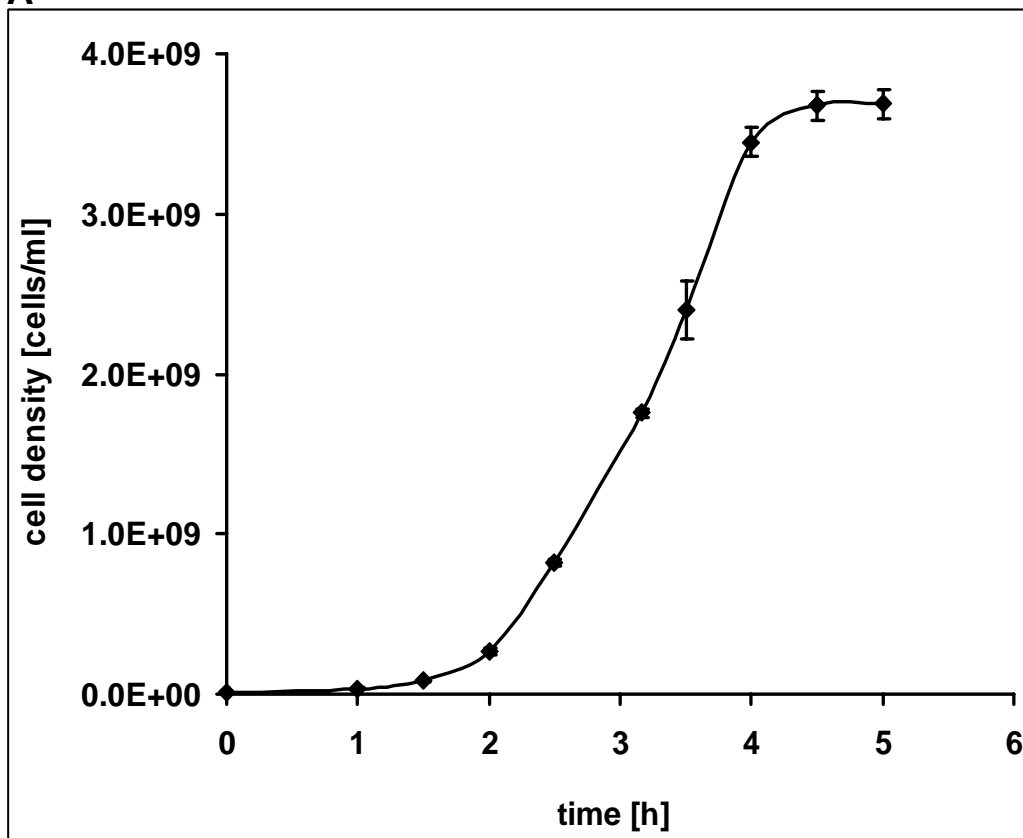**B**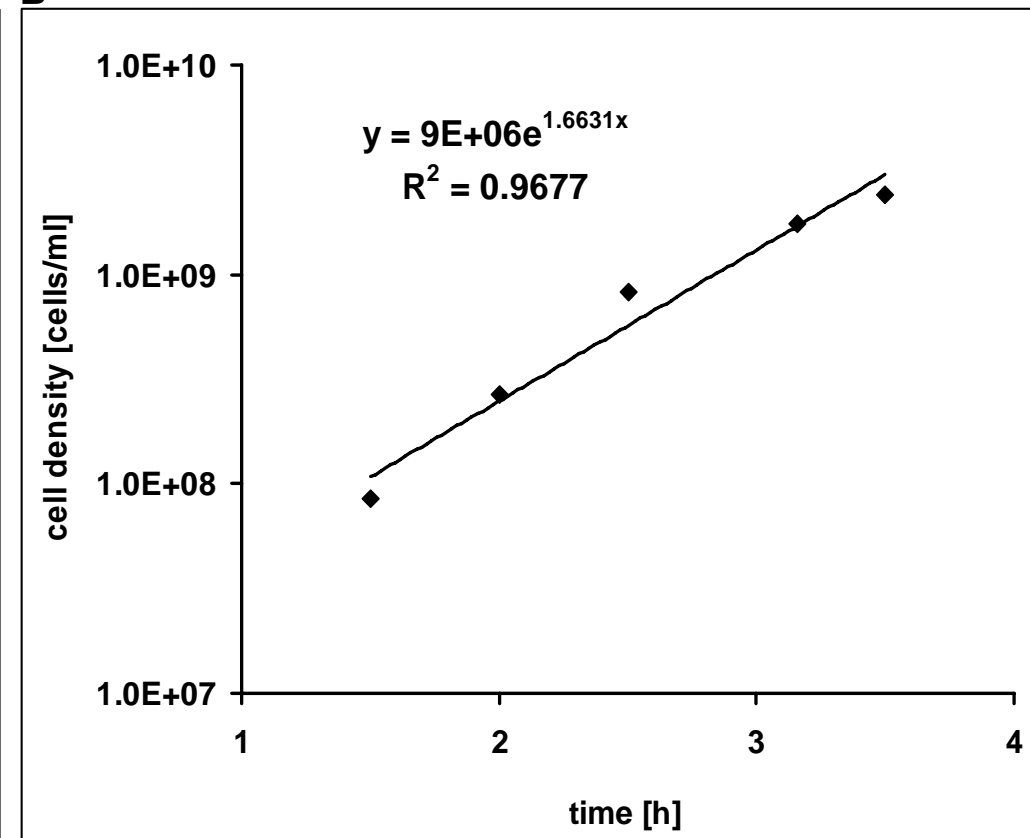

Supplement: Figure S1 — Growth of Escherichia coli in complex medium. A. Growth of E. coli in SOB+ complex medium. Samples for the determination of the genome copy number were taken in the exponential growth phase at a cell density of about 8×10^8^ cells/ml. B. Half logarithmic graph of the exponential growth phase. The line of best fit results in a doubling time of 25 min (PDF) [file pone.0016392.s001.pdf]

**A**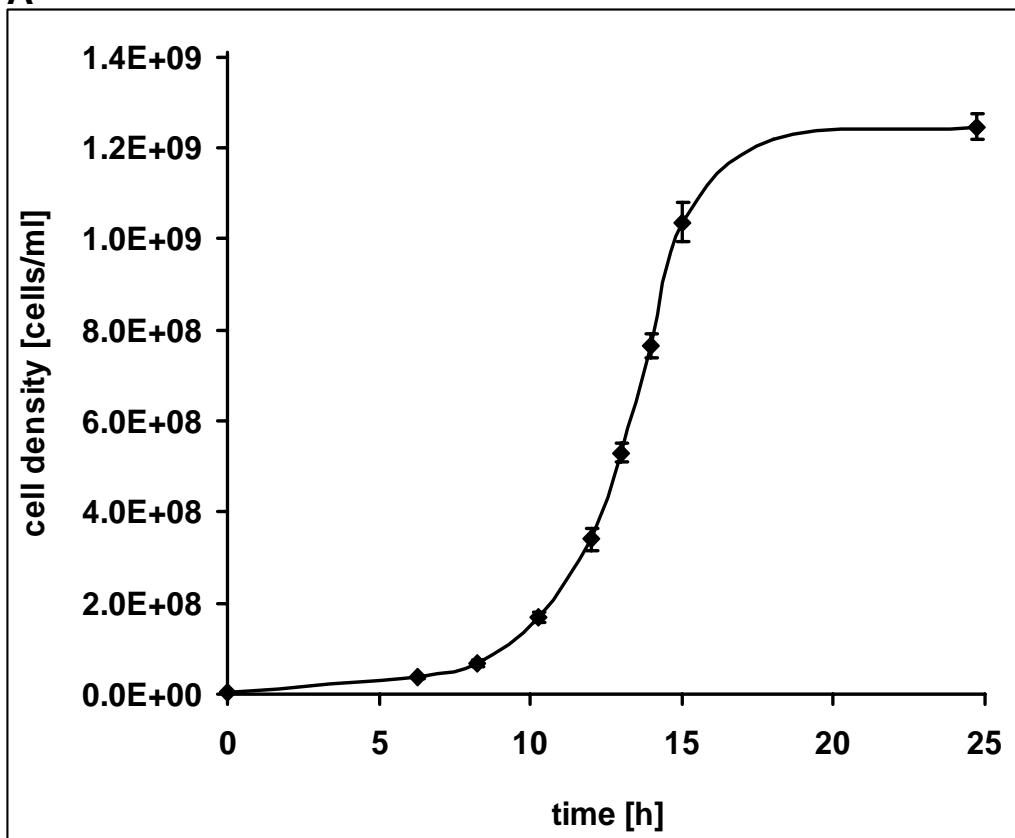**B**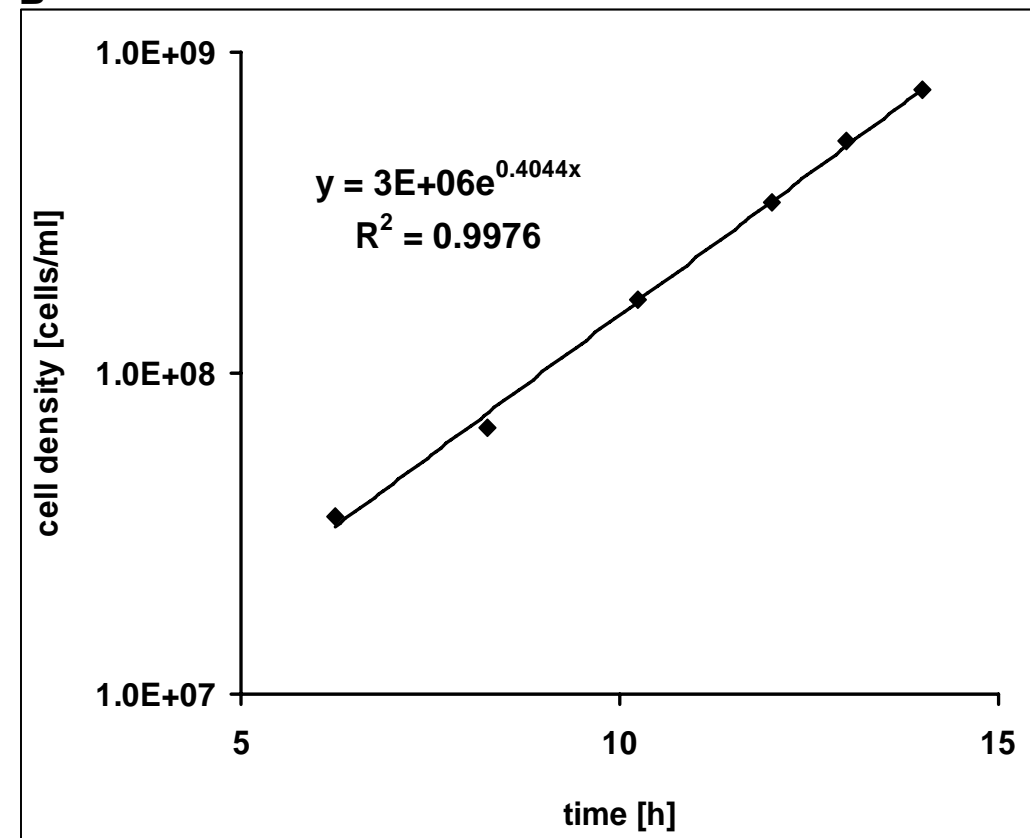

Supplement: Figure S2 — Growth of Escherichia coli in synthetic succinate medium. A. Growth of E. coli in M9 minimal medium with succinate as energy and carbon source. Samples for the determination of the genome copy number were taken in the exponential growth phase at a cell density of about 6×10^8^ cells/ml. B. Half logarithmic graph of the exponential growth phase. The line of best fit results in a doubling time of 103 min. (PDF) [file pone.0016392.s002.pdf]

**A**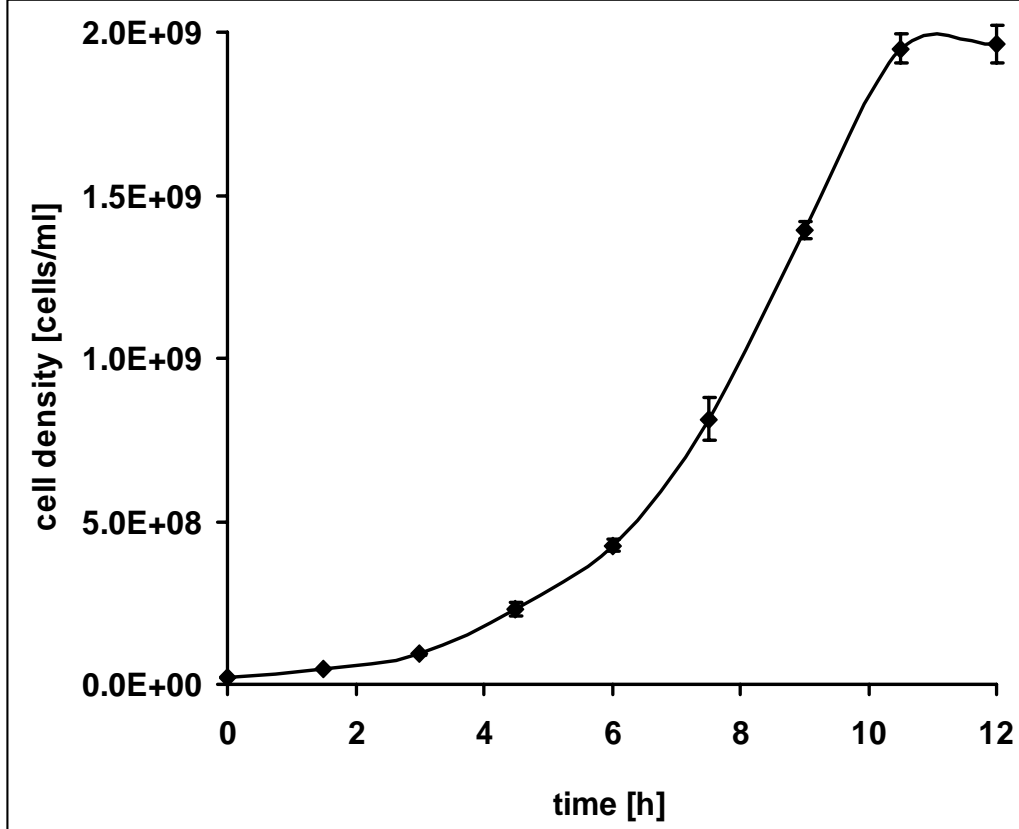**B**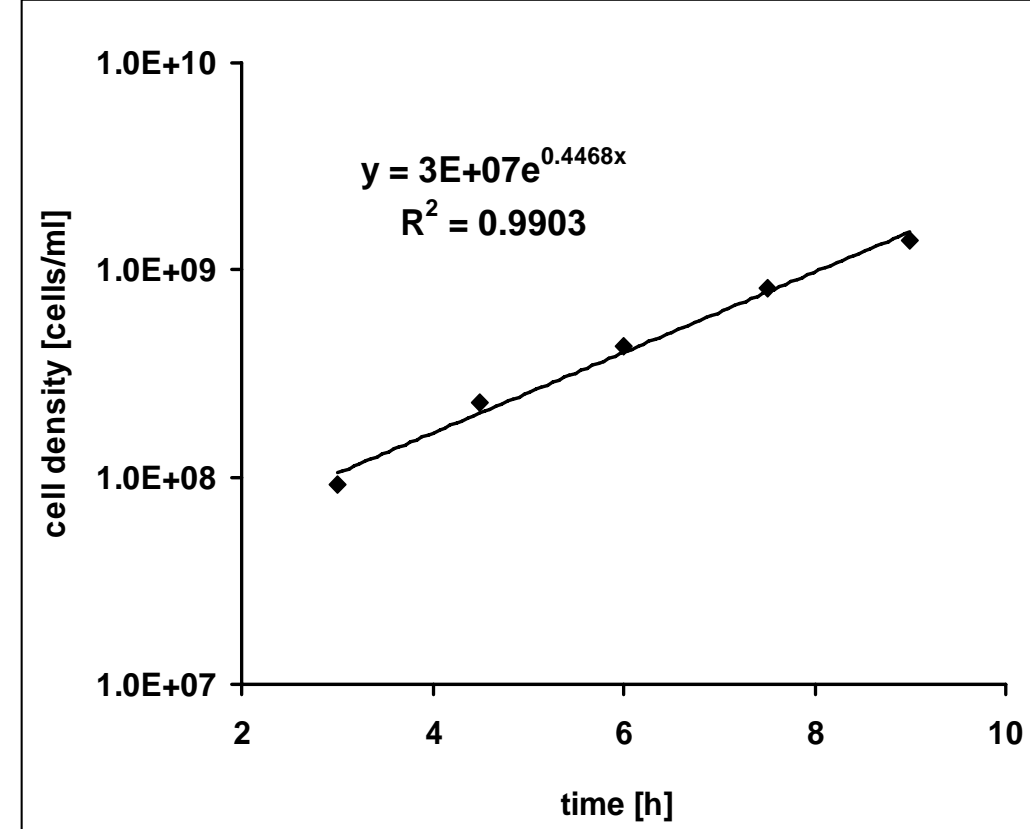

Supplement: Figure S3 — Growth of Caulobacter crescentus . A. Growth of C. crescentus in PYE complex medium. Samples for the determination of the genome copy number were taken in the exponential growth phase at a cell density of about 5×10^8^ cells/ml. B. Half logarithmic graph of the exponential growth phase. The line of best fit results in a doubling time of 93 min. (PDF) [file pone.0016392.s003.pdf]

**A**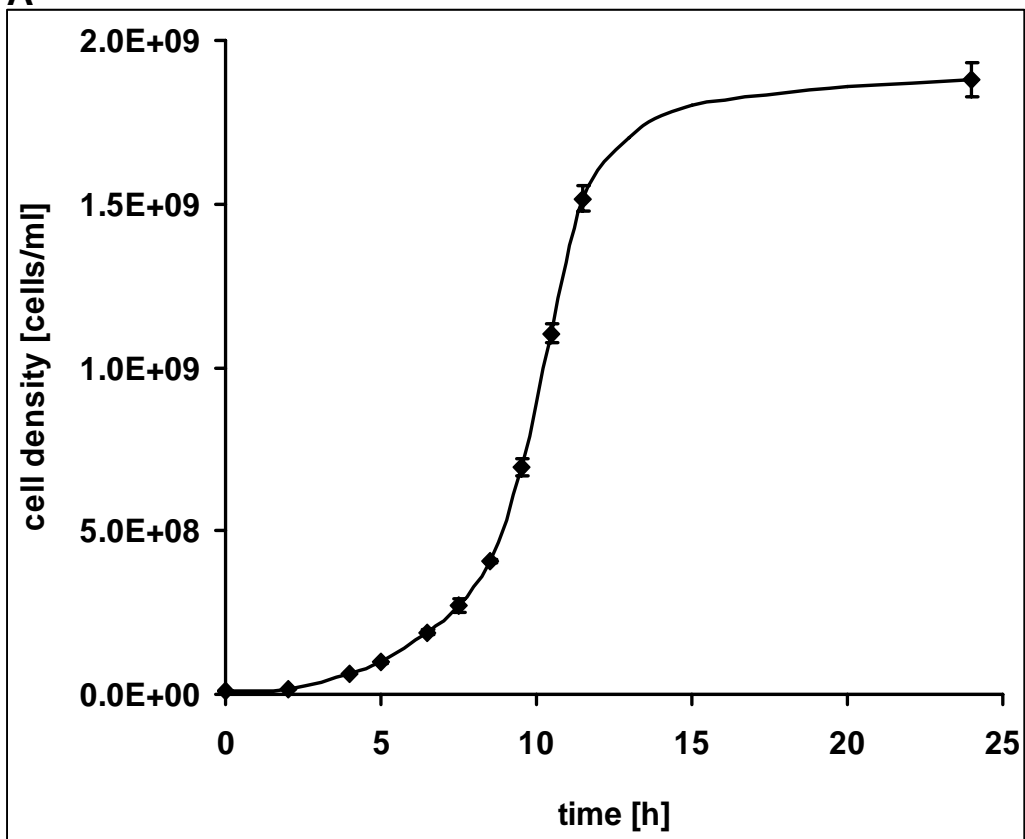**B**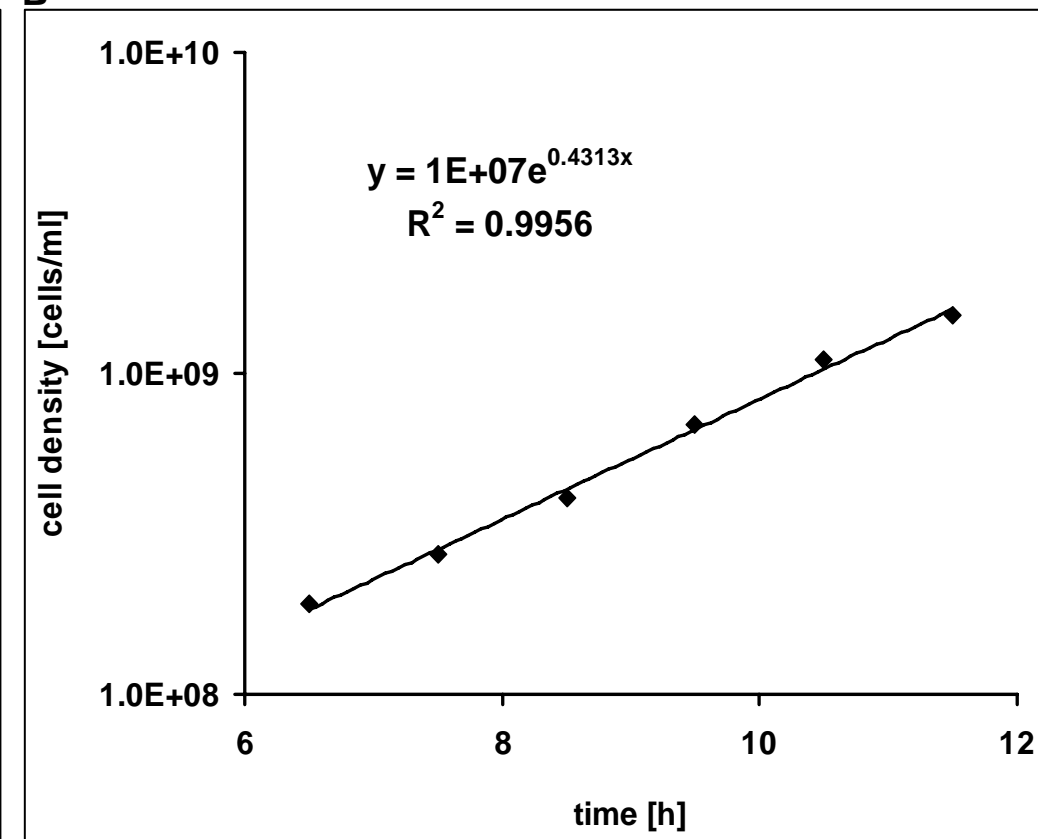

Supplement: Figure S4 — Growth of Wolinella succinogenes . A. Growth of W. succinogenes in fumarate medium. Samples for the determination of the genome copy number were taken in the exponential growth phase at a cell density of about 5×10^8^ cells/ml. B. Half logarithmic graph of the exponential growth phase. The line of best fit results in a doubling time of 96 min. (PDF) [file pone.0016392.s004.pdf]

**A**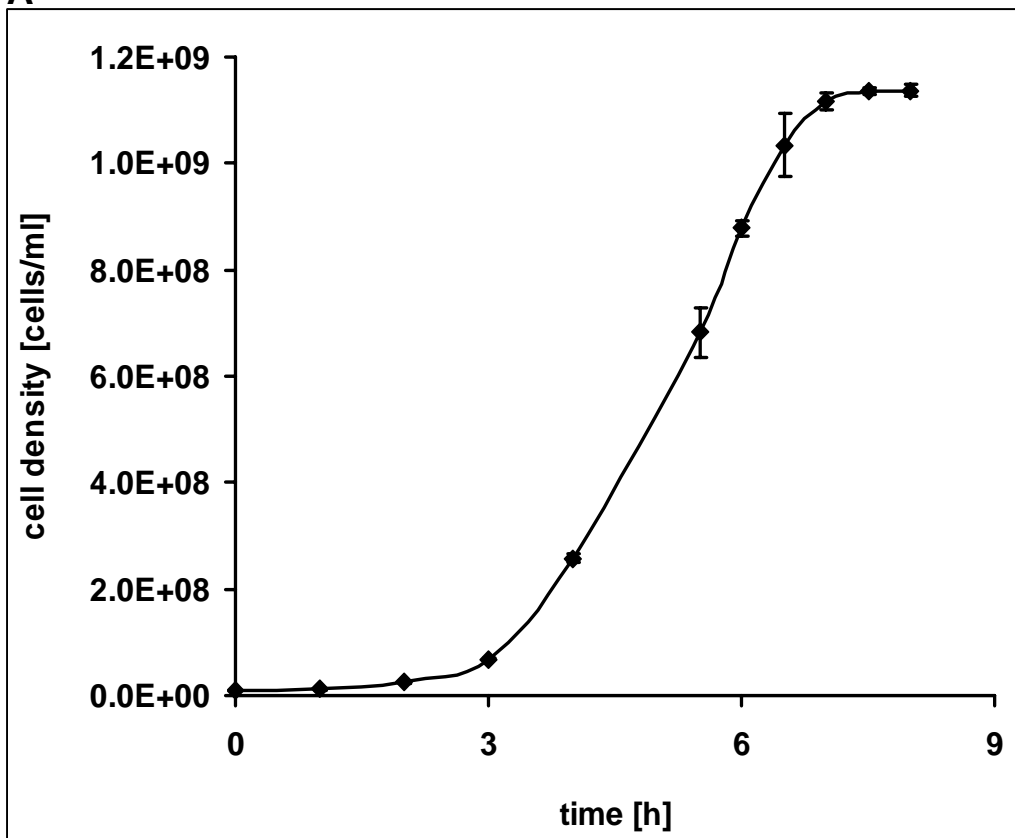**B**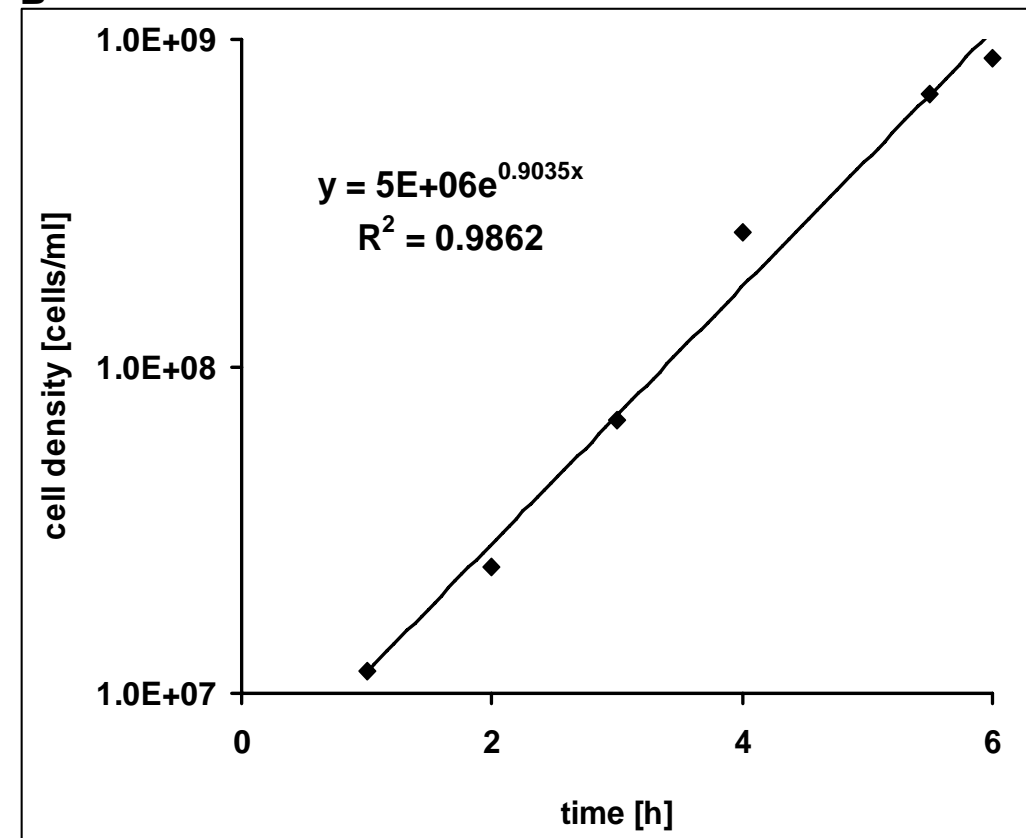

Supplement: Figure S5 — Growth of Pseudomonas putida . A. Growth of P. putida in Nutrient Broth complex medium. Samples for the determination of the genome copy number were taken in the exponential growth phase at a cell density of about 5×10^8^ cells/ml. B. Half logarithmic graph of the exponential growth phase. The line of best fit results in a doubling time of 46 min. (PDF) [file pone.0016392.s005.pdf]
